# An Unusual Presentation of Membranous Nephropathy in an Adult With Arthrogryposis: A Case Report

**DOI:** 10.7759/cureus.58339

**Published:** 2024-04-15

**Authors:** Upasana Agrawal, Manush Sondhi, Nitesh Gandhi, Shivani Sharma

**Affiliations:** 1 Department of Internal Medicine, Louisiana State University Health Sciences Center, Shreveport, USA

**Keywords:** nephrotic-range proteinuria, flank pain, hypercoagulable state, arthrogryposis multiplex congenita, primary membranous nephropathy

## Abstract

Membranous nephropathy (MN) is an autoimmune condition that is a common cause of nephrotic syndrome in nondiabetic adults. In this study, we highlight a case of a 22-year-old male with a past medical history of arthrogryposis multiplex congenita (AMC) who initially presented with right flank pain and hematuria. Subsequent workup revealed significant proteinuria with biopsy-proven primary MN. Early detection of the disease is critical to establish treatment promptly and prevent complications such as those resulting from a hypercoagulable state.

## Introduction

Membranous nephropathy (MN) is a condition affecting the glomeruli that can manifest at any stage of life. Among adults, it stands as the leading cause of nephrotic syndrome. Approximately 80% of MN cases occur without a clear underlying cause (referred to as primary MN). Conversely, the remainder is linked to factors such as medication use or concurrent conditions including systemic lupus erythematosus, hepatitis virus infection, or cancer [1]. Our case focuses on a 22-year-old man with arthrogryposis whose initial presentation included acute flank pain and hematuria. Further workup revealed proteinuria, and, eventually, a diagnosis of primary MN was made after a kidney biopsy. This case underscores the significance of recognizing both the clinical symptoms and pathological findings promptly to initiate treatment early and mitigate the potential complications related to MN.

## Case presentation

**Figure 1:**
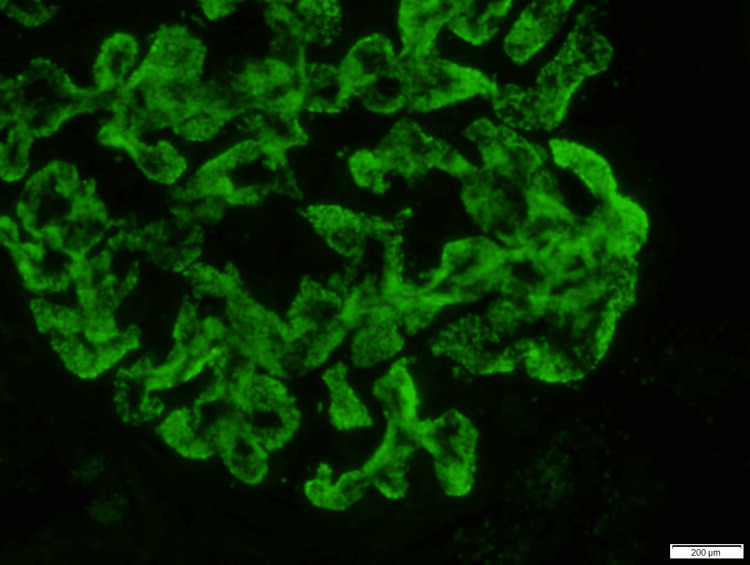
Immunofluorescence studies from the kidney biopsy: IgG4 and PLA2R stains of the glomeruli showed diffuse positive reactions along the capillary walls for IgG4 (2-3+) and weakly positive reaction to PLA2R (1-2+). These findings were suggestive of subepithelial immune complex deposition in the setting of primary membranous nephropathy

**Figure 2:**
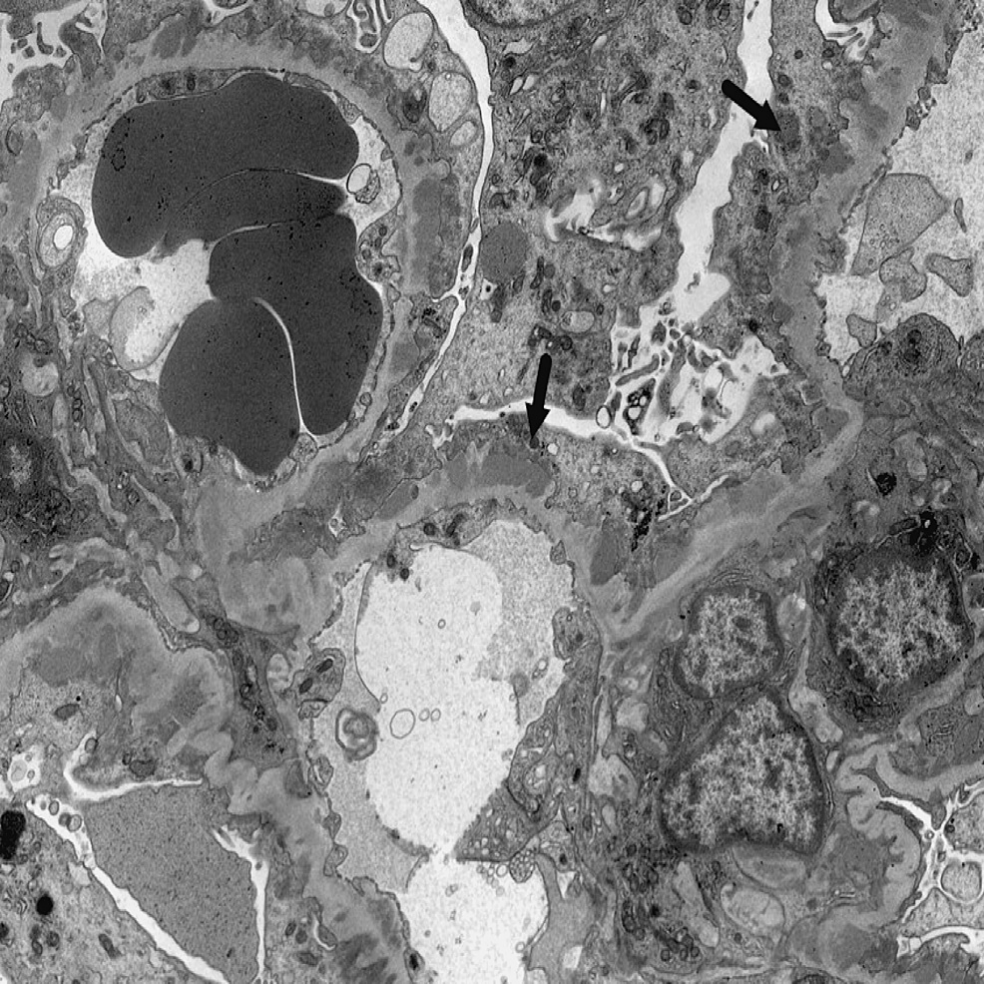
An electron microscopy revealed diffuse effacement of podocyte foot processes suggestive of membranous nephropathy

A 22-year-old male with arthrogryposis multiplex congenita presented to the emergency department (ED) with complaints of right flank pain and hematuria for one week. Flank pain was associated with radiation to the groin. The review of systems for other complaints was negative. Upon arrival at the ED, he was tachycardic, with a heart rate of 127 beats per minute. His other vital signs were stable. A physical examination revealed right costovertebral angle tenderness. The patient did demonstrate anasarca. Labs revealed a hemoglobin of 15.6 g/dL, WBC count of 6.77 K/uL, and platelets of 288 K/uL. The patient had hypoalbuminemia, with a serum albumin of 1.8 g/dL (normal range: 3.5-5.2 g/dL). Blood urea nitrogen (BUN) and creatinine levels were within normal levels. The hepatitis panel was negative. Test for antinuclear antibodies was nonreactive, and the complements C3 and C4 were within normal limits. Urinalysis and urine microscopy were remarkable for a proteinuria of 3+ and an RBC count of >100 per high power field (hpf). The urine protein-to-creatinine ratio was elevated to 7.87 (normal ratio: 0.00 to 0.20). Serum cholesterol levels were elevated to 255 mg/dL with a low-density lipoprotein (LDL) of 180 mg/dL. A CT renal stone study revealed mild wall thickening of the distended bladder, but it was negative for urolithiasis or hydroureteronephrosis bilaterally. Nephrology was consulted, and a kidney biopsy was recommended. Immunofluorescence studies from the kidney biopsy revealed subepithelial immune complex deposition, suggesting primary membranous glomerulopathy (Figure 1) with a weakly positive reaction (1 to 2+) to phospholipase A2 receptor (PLA2R) and a positive reaction (2 to 3+) to IgG4 staining. Electron microscopy revealed diffuse effacement of podocyte foot processes (Figure 2). Antibodies against serum phospholipase A receptor (PLAR) and thrombospondin type-1 domain containing 7A (THSD7A) were negative.

The patient was started on apixaban for the risk of hypercoagulability, rosuvastatin for hypercholesterolemia, and losartan for nephrotic syndrome. The patient was referred to rheumatology where treatment options including steroids and rituximab were discussed. Steroids were declined by the patient because of a long-standing history of muscle weakness and poor strength in the lower extremities. He was started on rituximab infusion but developed an allergic reaction to it that included a skin rash and throat swelling. The patient was initiated on tacrolimus, which was well tolerated. On the follow-up visits, the patient reported feeling symptomatically better. Labs also revealed a decrease in proteinuria from >10 grams per day at the time of diagnosis to <3 grams per day after the initiation of medical therapy.

## Discussion

MN occurs because of an autoimmune process characterized by subepithelial immune complex deposition. Primary MN is idiopathic, whereas secondary MN can occur because of infections, malignancy, and the use of certain drugs such as nonsteroidal anti-inflammatory drugs (NSAIDs), gold, and penicillamine. In almost 70% of patients with primary MN, autoantibodies to PLA2R are positive [2]. Our patient had negative serum PLA2R antibodies; however, the kidney biopsy was positive for PLA2R and IgG4 antibodies. Studies have discovered that the glomerular deposits are typically IgG1 dominant rather than IgG4 dominant in the very early stages (pure stage I) of MN. There are two reasons why this phenomenon might occur. While IgG4 antibodies are typically linked to polysaccharide antigens, IgG1 antibodies are typically induced in response to protein antigens. Thus, antigens other than PLA2R may play a pathogenic role in the early stages of primary MN. However, as the disease progresses, the PLA2R antibody response grows, and an anti-PLA2R IgG4 response eventually takes precedence [3]. Additionally, it is possible that as primary membranous glomerulonephritis (MGN) accelerates, there will be a switch in the IgG subclass. There may be an IgG1-dominant anti-PLA2R response in the early stages, which will likely change into an IgG4-dominant antibody response as the disease progresses. Such an IgG subclass switch has been linked to several illnesses, including paracoccidioidomycosis [4], leprosy, tuberculosis [5], and malaria [6]. Other studies have also focused on IgG4-related diseases affecting the kidneys. Tubulointerstitial nephritis (IgG4-related tubulointerstitial nephritis (TIN)) is a well-known manifestation of IgG4-RD that can manifest as renal failure, a mass-like lesion, or both. MGN, membranoproliferative glomerulonephritis, mesangioproliferative immune complex glomerulonephritis, and IgA nephropathy/Henoch-Schönlein purpura are the specific glomerular diseases that have been reported. MGN is the most described glomerular disease in IgG4-RD kidney tissue from idiopathic membranous nephropathy (IMN) patients who had a positive rate of IgG4 expression of 81.48%. Conversely, kidney tissue from secondary membranous nephropathy (SMN) and non-MN patients had positive rates of 20% and 14.9%, respectively [7].

Arthrogryposis or arthrogryposis multiplex congenita (AMC) is a descriptive term for the development of nonprogressive contractures affecting one or more areas of the body before birth. After a review of the literature, no evidence could be found on the association of arthrogryposis with primary membranous glomerulopathy. However, an autosomal-recessive disorder called arthrogryposis, renal dysfunction, and cholestasis (ARC) syndrome is caused by mutations in the VPS33B and VIPAR genes. However, usually, these children do not survive past one year, and mortality is usually secondary to infectious complications. Proximal tubular dysfunction is the traditional renal manifestation of ARC syndrome that has been previously documented [8].

Moreover, our patient had an unusual clinical presentation with flank pain and hematuria, with no radiographic evidence of urolithiasis or hydroureteronephrosis, but he was subsequently diagnosed with membranous glomerulopathy based on the kidney biopsy. A case of a 58-year-old male has been reported where the patient presented with acute flank pain. However, he was diagnosed with nephrotic syndrome because of biopsy-proven idiopathic MN. Later, a CT angiogram of the abdomen revealed a right renal vein thrombosis, which was treated with thrombectomy and local thrombolytic therapy [9]. This indicates the need to consider hypercoagulability because of proteinuria in such patients. When it comes to the disease outcome, a third of patients achieve remission, a third are stable, and a third have persistent proteinuria and progressive kidney damage [2]. Cyclophosphamide plus corticosteroids are effective immunosuppressive agents in preventing progression in patients at high risk for kidney failure. Given the possibility of carcinogenic side effects, therapies involving calcineurin inhibitors and CD20-targeted B cell depletion therapies, including rituximab, have been developed. However, it has been studied that in patients with a high titer of PLA2R antibody, rituximab has a lower remission rate than cyclophosphamide. In addition to these existing drugs, antigen-specific immunotherapies are currently being developed [1].

## Conclusions

In conclusion, this case underscores the importance of considering MN as a potential etiology in young adults presenting with flank pain, hematuria, and significant proteinuria. Prompt recognition and diagnosis are paramount in initiating timely treatment to mitigate the risk of complications associated with this condition, particularly the hypercoagulable state that can lead to thromboembolic events. Clinicians should maintain a high index of suspicion for MN in the differential diagnosis of nephrotic syndrome in nondiabetic adults, facilitating early intervention and improved patient outcomes. Further research into the underlying pathophysiology and therapeutic strategies for this condition is warranted to enhance management strategies and optimize patient care.

## References

[1] Ronco P, Beck L, Debiec H (2021). Membranous nephropathy. Nat Rev Dis Primers.

[2] Fogo AB, Lusco MA, Najafian B, Alpers CE (2015). AJKD atlas of renal pathology: membranous nephropathy. Am J Kidney Dis.

[3] Huang CC, Lehman A, Albawardi A (2013). IgG subclass staining in renal biopsies with membranous glomerulonephritis indicates subclass switch during disease progression. Mod Pathol.

[4] Baida H, Biselli PJ, Juvenale M, Del Negro GM, Mendes-Giannini MJ, Duarte AJ, Benard G (1999). Differential antibody isotype expression to the major Paracoccidioides brasiliensis antigen in juvenile and adult form paracoccidioidomycosis. Microbes Infect.

[5] Sousa AO, Henry S, Marója FM (1998). IgG subclass distribution of antibody responses to protein and polysaccharide mycobacterial antigens in leprosy and tuberculosis patients. Clin Exp Immunol.

[6] Tongren JE, Drakeley CJ, McDonald SL (2006). Target antigen, age, and duration of antigen exposure independently regulate immunoglobulin G subclass switching in malaria. Infect Immun.

[7] Alexander MP, Larsen CP, Gibson IW (2013). Membranous glomerulonephritis is a manifestation of IgG4-related disease. Kidney Int.

[8] Holme A, Hurcombe JA, Straatman-Iwanowska A, Inward CI, Gissen P, Coward RJ (2013). Glomerular involvement in the arthrogryposis, renal dysfunction and cholestasis syndrome. Clin Kidney J.

[9] Perazella MA (2020). A patient with nephrotic syndrome and acute flank pain. Kidney360.

